# Detection of *Torque Teno Virus* (TTV) and *TTV-Like Minivirus* in patients with presumed infectious endophthalmitis in India

**DOI:** 10.1371/journal.pone.0227121

**Published:** 2020-01-07

**Authors:** Poonam Naik, Vivek Pravin Dave, Joveeta Joseph

**Affiliations:** 1 Jhaveri Microbiology Centre, Brien Holden Eye Research Centre, L. V. Prasad Eye Institute, Hyderabad, India; 2 Research Scholar, Manipal Academy of Higher Education, Manipal, India; 3 Smt. Kanuri Santhamma Centre for Vitreo-Retinal Diseases, L. V. Prasad Eye Institute, Hyderabad, India; Universita degli Studi di Parma, ITALY

## Abstract

Human anelloviruses (Torque Teno Virus (TTV) and TTV Like Mini Virus (TLMV)) are now being reported at a high prevalence across the world, with a controversial disease-inducing potential. The aim of this study was to investigate the role of these anellovirus in vitreous of patients with presumed infectious endophthalmitis. After informed consent, vitreous fluid from patients with endophthalmitis (*n* = 103) and non-infectious pathologies (*n* = 102) were analyzed for the presence of TTV and TLMV DNA by qPCR with the limit of quantification defined as 100 copies per reaction. Among the patients clinically diagnosed with endophthalmitis, 29 of the 40 culture proven samples (72.5%) and 42 out of 63 (66.6%) of culture-negative samples were positive for presence of TTV/TLMV. Interestingly, 51 of the 102 (50%) samples in the control group were also positive for TTV/TLMV. Comparing the clinical outcome among patients diagnosed with endophthalmitis, we observed no significant association in the final visual acuity of patients who were positive for presence of TTV/TLMV, however, these patients had significantly higher repeat antibiotic injections (p = 0.03). Further evidence is however needed to correlate TTV / TLMV with a particular pathology or group of pathologies in the eye.

## Introduction

Endophthalmitis is a serious intraocular inflammation caused usually by bacterial or fungal infection. Most cases are medical emergencies, and delay in administration of treatment may result in permanent loss of vision [1]. The ability to identify the causative pathogen(s) has wide implications in clinical management of these patients. Wide variation from 38–44% in positivity of microbiology culture in presumed infectious endophthalmitis cases is reported [2–4] and the etiologic organism is thus undiagnosed in a large number of patients. Recent reports of identification of *Torque Teno Virus* in the vitreous humor of patients with panuveitis and culture negative endophthalmitis [5,6], has suggested that *Anellovirus* might have a role in the pathogenesis of endophthalmitis, thereby suggesting a detailed investigation on the possible role of TTV in the pathogenesis of endophthalmitis in different populations. Human anelloviruses, namely *Torque teno virus* (TTV), and *TTV-like mini virus* (TLMV), species are characterized by their staggering worldwide prevalence, with conflicting opinions on their pathogenic role in the literature. [7–9] Several reports on TTV have demonstrated that these viruses are commonly present circulating in the blood and other tissues in patients with chronic hepatitis, cirrhosis, and hepatocellular carcinoma [7–9], for extended periods or throughout their lifetime. But none of the reports have found TTV as a cause of acute and/or chronic liver diseases. [10] At present, however, no human disease has been confirmed to be linked with TTV, which is reported to be the most abundant virus of the human microbiome. [11]

A previous report by Emre et al. [12] reported the occurrence of TTV DNA in aqueous humour specimens of patients without any other ocular pathology, and found TTV by real time PCR in 3 (12.5%) of the 24 specimens only. While there still are contradicting reports on the pathogenic role of TTV owing of its high prevalence in the blood of the general population world over, the pathological mechanisms of anelloviruses are however not fully understood and recent literature only highlights the possible immunomodulatory effect of TTV [13]. While there are many studies correlating the presence of these viruses with inflammation, there are no reports that aim to identify a link between the prevalence of human anelloviruses and endophthalmitis, as well as other ocular pathologies (i.e. diabetic retinopathy, retinal detachment and AMD). Also, the presence of TTV and /or TLMV in the Indian population has not been assessed before. Thus, the study aimed to estimate the presence of anellovirus in the vitreous fluid of patients with presumed infectious endophthalmitis and compare with other ocular pathologies in the Indian population and to understand its clinical significance.

## Material and methods

### Vitreous samples

A total of 103 vitreous samples from patients who presented to the institute between December 2017 and June 2018 and diagnosed clinically as infectious endophthalmitis, after vitrectomy were included in the study group. Similarly the control group included 102 samples from patients undergoing vitrectomy for non-infectious retinal disorders. Informed consent was obtained from all patients and approved by the Institutional Review Board, L V Prasad Eye Institute, Hyderabad, India. The microbiology work up of the samples from the study group was performed as described previously. [14] Approx. 200μl of the samples were stored at -20°C for molecular analysis.

### Anellovirus detection and genetic characterization

DNA was extracted from the vitreous samples using the QIAamp DNA minikit (Qiagen, Germany). Presence of both TTV and TLMV was then assessed by q-PCR using SYBR green method to detect the presence of TTV and TMLV in all samples, on the Quant studio 3 (Applied Biosystems) system. The primers for q-PCR were chosen to amplify a distinct conserved region of TLMV upstream of the ORF 2, and within the ORF2 in TTV. [15,16] β-Actin served as the positive control in every assay. In all the q-PCR reactions, standards with known copies of TTV and TLMV, and no template controls were additionally included and samples were tested in duplicate. Following amplification, melt curve analysis was performed to confirm the specificity of the PCR product. The experimental conditions for the dissociation curve analysis were 95°C for 15 seconds followed by 60°C for 1 minute and then 95°C for 15 seconds with a ramp rate of +0.3°C/second. The specificity of the PCR products were also re-confirmed by a nucleotide BLAST analysis of the sequenced amplicons. The patients were also analyzed by chi-square test, based on their final Visual Acuity and clinical outcome based on the presence or absence of these allenovirus. The medical records of these patients were further analyzed for the number of repeat surgical intervention and the level of significance was calculated as a P < 0.05. The statistical analyses were performed with SPSS software (v 16.0).

## Results

The study included 103 vitreous samples in the infectious group and 102 samples in control group of which the mean age was 50.54 ± 16.23 years (range 9–77 years) in the control group and 42.44 ± 21.95 years (range 1–88 years) in the study group, respectively. These included 68 (66.6%) males and 34 (33.4%) females in the control group and 75 (72.8%) males and 28 (27.2%) females in the study group. The clinical diagnosis of patients diagnosed with infectious endophthalmitis included post-trauma in 50 (48.5%) and post-operative in 29 (28.1%) while 15 patients (14.5%) were diagnosed as endogenous endophthalmitis (Table 1).

**Table 1:** Details of distribution of demographic details and clinical diagnosis of patients included in the study and control group.

Variable	Controls N = 102	Study group—infectious cases N = 103
**Age (years) Mean ± SD Range**	50.54 ± 16.23 (9–77)	42.44 ± 21.95 (1–88)
**Gender**		
**Male**	68 (66.6%)	75 (72.8%)
**Female**	34 (33.3%)	28 (27.2%)
**Diagnosis**	RD-37 (36.2%)	Post—Operative—29 (28.1%)
	MH– 10 (9.8%)	Post-traumatic—50 (48.54%)
	PDR– 25 (24.5%)	Endogenous– 15 (14.5%)
	Others– 30 (29.4%)	MK associated– 9 (8.7%)

RD: Retinal Detachment, MH: Macular hole, PDR: Proliferative Diabetic Retinopathy; MK: Microbial Keratitis.

**Fig 1:**
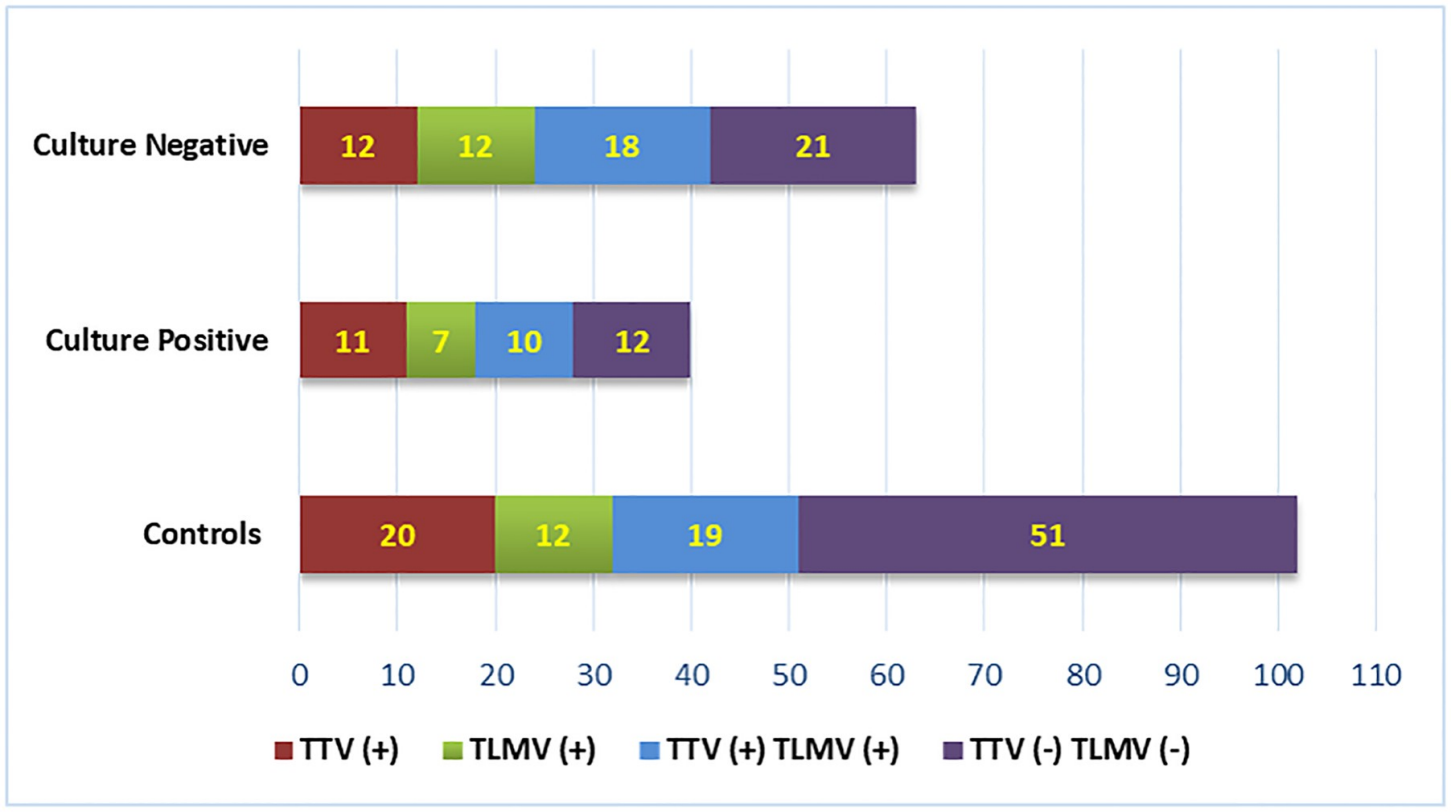
Histogram showing detection of TTV and / or TLMV DNA in vitreous of patients in the control group, culture positive and culture negative group.

Of these 103 presumed infectious samples, 63/103 (61.1%) samples were culture negative while 40/103 (38.9%) samples were culture positive and included bacterial growth in 35/40 (87.5%) while 5/40 (12.5%) grew fungus. Human anellovirus was detected in 71/103 (68.9%) infectious samples which included TTV in 23/103 and TLMV in 20/103 samples while 28/103 samples showed presence of both TTV and TLMV. Comparatively, 51/102 (50%) samples in the control group also showed presence of TTV and/ or TLMV in the vitreous fluid (Fig 1).

**Fig 2:**
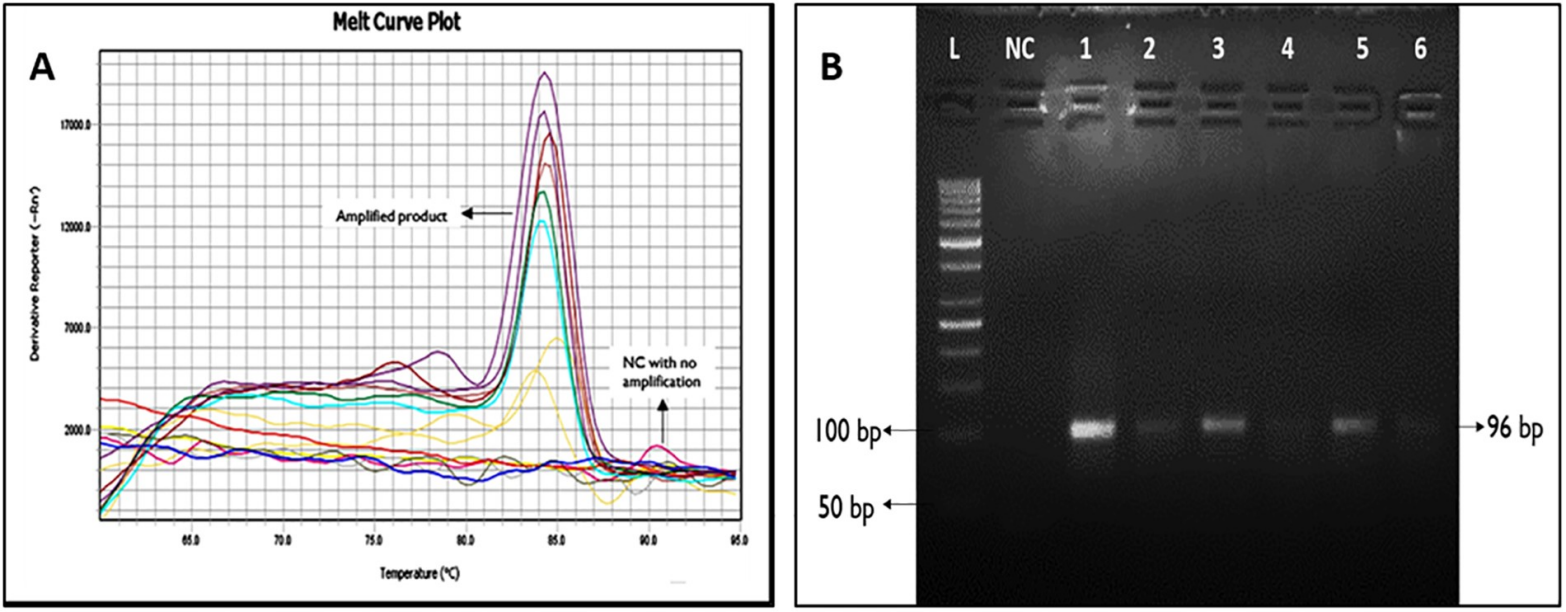
A: Melting curve analysis of TTV and / or TLMV real-time PCR products: the figure shows the specific melting temperature of human TTV and/ or TLMV PCR products. Negative control and allenovirus negative samples showed no amplification with similar melt curve pattern. (B) TTV detection by electrophoresis after qPCR on agarose gel. Lane 1–6: patients with TTV infection; lane NC: Negative Control; lane L: 50 bp ladder.

The melting curve (melting temperature 84.04 ± 0.24 (mean ± SD) (Fig 2A) analysis confirmed the specificity of the TTV and TLMV specific 96 bp amplicon in all the samples (Fig 2B). The viral load ranged from 100 copies/reaction to 10x 10^5^ copies /reaction among all the groups tested and there was no significant correlation between copy number and culture proven infection.

Comparing the two groups, we found a significant association of anellovirus with endophthalmitis (OR = 2.32, 95% CI = 1.31–4.12, p = 0.006). No significant difference was observed in the distribution of TTV and/or TLMV in men and women participating in this study. Also the mean age, and clinical diagnosis were similar in TTV/TLMV positive and TTV/TLMV negative patients. Comparing detection of TTV and/or TLMV in culture negative and culture positive samples in the presumed infectious endophthalmitis group, we observed presence of TTV and/or TLMV in 29/40 (72.5%) culture positive samples and in 42/63 (63.4%) culture negative vitreous samples. This association of TTV and/or TLMV as a co-infection in patients with culture positive endophthalmitis was however not significant (*p* = 0.532) compared to patients with culture negative endophthalmitis. The visual outcome after two months of follow up was further assessed in patients that showed presence of anellovirus and it was found that 49/71 (69%) patients diagnosed with infectious endophthalmitis had unfavorable (>20/200) visual outcomes compared to patients who did not show presence of these viruses, although the difference was not statistically significant (*p* = 0.56). Further we investigated the number of surgical interventions required or attempted in all patients and compared it with the presence or absence of allenovirus. We observed that patients who were TTV and /or TLMV positive patients had significantly higher repeat antibiotic injections (p = 0.03) as shown in Table 2. However, there was no significant clinical difference either in the visual outcome or number of surgical interventions in the patients with both TTV and TLMV infection than those with just single TTV or TLMV infection.

**Table 2:** Comparison of the clinical outcome in patients diagnosed clinically with endophthalmitis and who showed presence or absence of TTV and/or TLMV.

Clinical outcome		TTV and/or TLMV (+ve)	TTV and/or TLMV (-ve)	*p* value
**No. of Surgical interventions**	>2	35	8	**0.03**
	<2	37	23	
**Final Visual acuity**	<20/200	32	17	0.56
	>20/200	11	8	

## Discussion

The discovery of TTV and its variants as a ubiquitous virus in human body has generated a lot of interest in the medical literature especially because it has been now reported to be the most representative and abundant virus of the human virome. Increasing evidence is accumulating that these group of anelloviruses contribute to the interplay of immune factors and/or are involved in the immune balance. [17] It is also being reported that imbalance of immune status affects TTV replication. [18–20] The present study offers further information on this matter and profiles the detection of anelloviral DNA in the vitreous of patients diagnosed clinically as endophthalmitis as 68.9%. Data in our study also show presence of higher levels of anelloviruses DNA in control samples from non-infectious patients as well, which is contradictory to the study by Lee at al. [5] These results may indicate a possible stratification of TTV frequency in the Indian population. TTV is reported to have several routes of transmission, including blood [7] so various intra-ocular surgical procedures could be potential gateways for TTV/TLMV to gain access into the blood stream. Though there no studies to support this hypothesis, in our study significantly higher rates of anelloviral DNA were found in subjects with retinal detachment and diabetic retinopathy. Previous studies have reported that the retinal pigment epithelium shields the sensitive neural retina from pathogens circulating in the bloodstream, and therefore disruption of that barrier due to diabetes or endogenous endophthalmitis, result in Blood Retinal Barrier breakdown and increases the likelihood of bacterial migration from the blood into the interior of the eye. [21, 22] Additionally Jung *et al*. [23] have earlier reported that ocular infections occurred in 9% of the patients with *S*.*aureus* bacteremia, and only 30% of those had diabetes as an underlying condition. Thus an intriguing hypothesis that the present study suggests that the environment created by a compromised blood retinal barrier resulting from endophthalmitis or vitreous hemorrhage promoted the entry of blood borne pathogens into the eye. This hypothesis that TTV could have originated from the systemic pool could have been validated if we collected paired serum and vitreous samples and corroborated the detection of the virus. In another study [6], anelloviruses was observed in vitreous of patients with uveitis, but not in patients with retinal detachment, and the authors hypothesized that it most likely originated from the bloodstream after inflammation-induced disruption of the blood-ocular barrier. However unfortunately, we were unable to perform this validation as only vitreous samples, were collected as part of microbiological diagnosis and TTV testing and clinical data analysis was done retrospectively and is one of the limitations in this study.

Another interesting finding of the study was that most vitreous specimens in both control and infectious group were simultaneously infected with more than one virus of the anellovirus group. The association of TTV or TLMV in different ocular conditions in the study support the theory that human anelloviruses have different biological role in the pathogenesis of various ocular conditions. This fact is strengthened by the observation that the patients who were positive for anellovirus required increased number of intravitreal antibiotic injections as compared to those patients who were negative for these viruses. While it may not be involved in causation of a disease or condition in the eye, it might however be acting as a bystander without much impact of its single or co-infection with other viruses. Recently, a case of human infectious endophthalmitis caused by Pseudorabies virus after exposure to sewage water in a hog farm in China suggesting that viruses are now being recognized as etiologic agents in endophthalmitis. [24] These results are preliminary and further larger studies are necessary to understand the true pathological significance of TTV/TLMV in endophthalmitis. It would also be interesting to analyze repeated samples over a period of time for possible fluctuations of TTV in vitreous of patients with endophthalmitis and study the exact clinical implications of presence of these viruses. Taken together, the results in this study provide, for the first time, a novel insight into an intra-host transmission of TTV and its prevalence in the eye.

## Conclusions

While TTV and TLMV appeared to be associated with increased severity and infection, it does not seem to be unequivocally associated with endophthalmitis but might have a pathological consequence. Our findings revealed for the first time the circulation of TTV and / or TLMV strains among Indian population with remarkably high infection rates in all the groups included in the investigation.

## Supporting information

S1 Fig TTV DNA detection by electrophoresis after qPCR on agarose gel captured (Raw image without contrast adjustment) at the bottom of the sample loaded lanes by Bio-Rad’s Gel Doc XR. Lane 1–50 bp ladder, Lane 2- Negative Control, Lane 3–8: patients with TTV infection (96 bp).
(PDF)
