# Supplementary material for: Detection of Torque Teno Virus (TTV) and TTV-Like Minivirus in patients with presumed infectious endophthalmitis in India
Source: PLoS One. 2020 Jan 7;15(1):e0227121. doi: 10.1371/journal.pone.0227121 (PMC6946165; doi:10.1371/journal.pone.0227121)
Supplement: S1 Fig — Lane 1–50 bp ladder, Lane 2- Negative Control, Lane 3–8: patients with TTV infection (96 bp). (PDF) [file pone.0227121.s001.pdf]

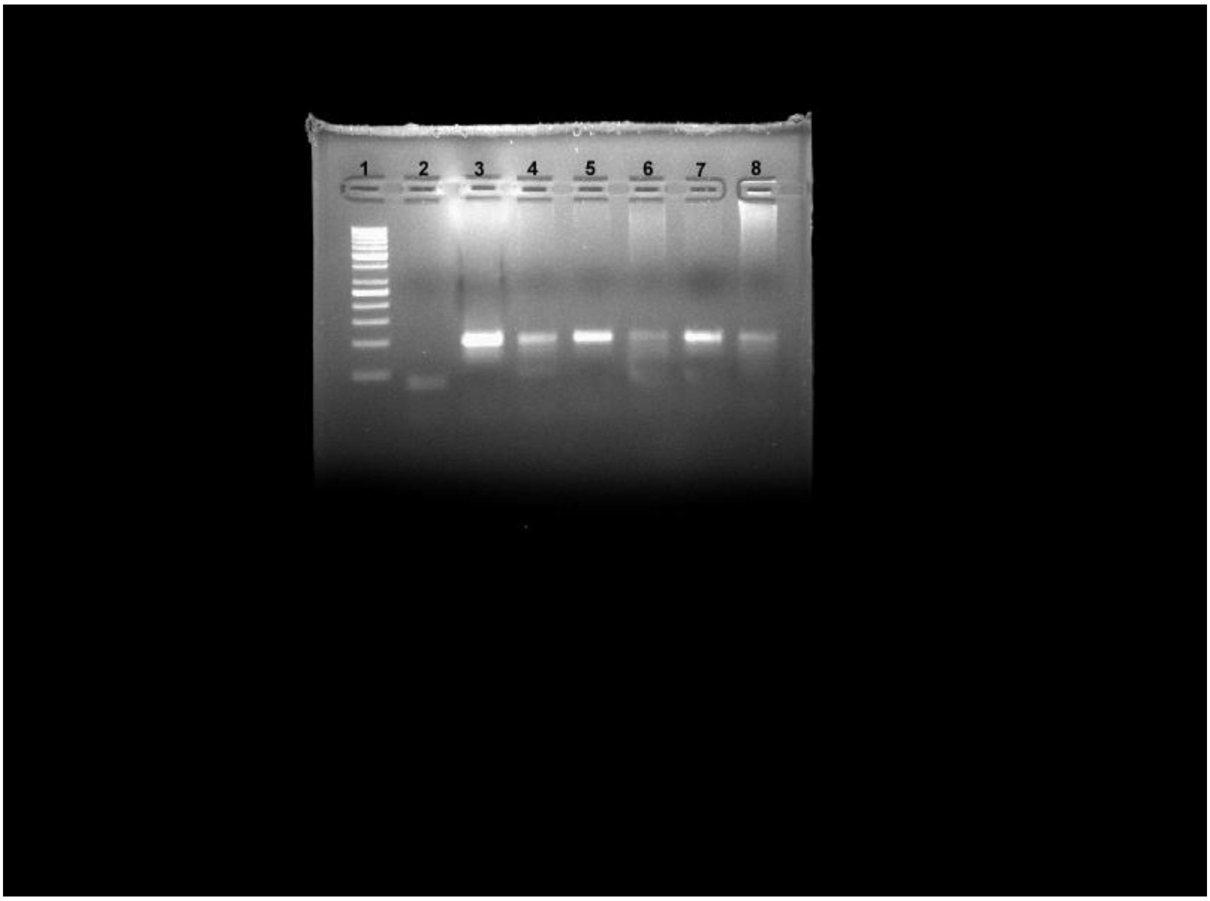

Figure 2B: TTV DNA detection by electrophoresis after qPCR on agarose gel captured (Raw image without contrast adjustment displaying faint primer dimers (less than 50bp size) at the bottom of the sample loaded lanes by Bio-Rad's Gel Doc XR. Lane 1- 50 bp ladder, Lane 2- Negative Control, Lane 3-8: patients with TTV infection.
